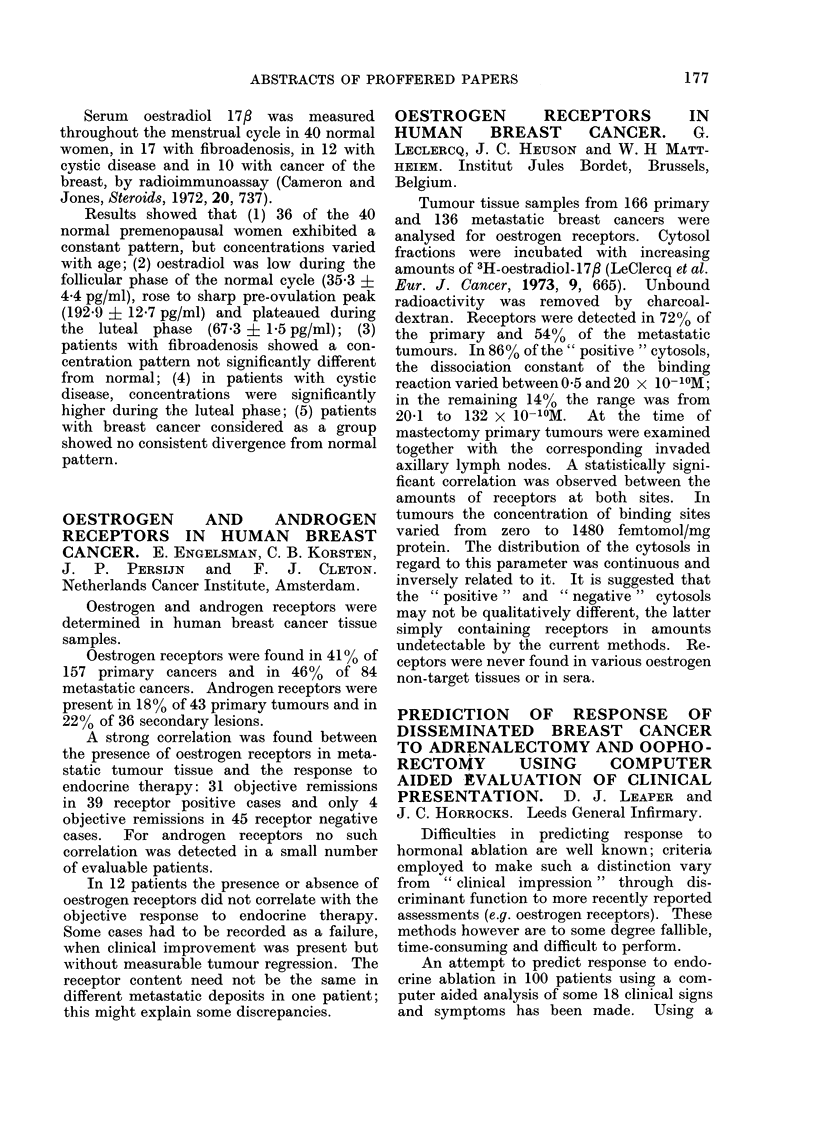# Proceedings: Oestrogen receptors in human breast cancer.

**DOI:** 10.1038/bjc.1974.146

**Published:** 1974-08

**Authors:** G. Leclercq, J. C. Heuson, W. H. Mattheiem


**OESTROGEN RECEPTORS IN HUMAN BREAST CANCER.** G. LECLERCQ, J. C. HEUSON and W. H MATTHEIEM. Institut Jules Bordet, Brussels, Belgium.

Tumour tissue samples from 166 primary and 136 metastatic breast cancers were analysed for oestrogen receptors. Cytosol fractions were incubated with increasing amounts of $^3$H-oestradiol-17$\beta$ (LeClercq *et al. Eur. J. Cancer*, **1973**, **9**, 665). Unbound radioactivity was removed by charcoal-dextran. Receptors were detected in 72% of the primary and 54% of the metastatic tumours. In 86% of the " positive " cytosols, the dissociation constant of the binding reaction varied between 0·5 and 20 $\times$ $10^{-10}$M; in the remaining 14% the range was from 20·1 to 132 $\times$ $10^{-10}$M. At the time of mastectomy primary tumours were examined together with the corresponding invaded axillary lymph nodes. A statistically significant correlation was observed between the amounts of receptors at both sites. In tumours the concentration of binding sites varied from zero to 1480 femtomol/mg protein. The distribution of the cytosols in regard to this parameter was continuous and inversely related to it. It is suggested that the " positive " and " negative " cytosols may not be qualitatively different, the latter simply containing receptors in amounts undetectable by the current methods. Receptors were never found in various oestrogen non-target tissues or in sera.